# Book Reviews

**Published:** 1846-06

**Authors:** 


### Bibliographical Notices.

*Nouveaux Eléments Complets de la Science, et de L'Art Du Dentiste,* Par (M.) Desirabode, *Chirurgien Dentiste* Du Roi, *et* Ses Fils, *Docteurs en Médecine;* Suivis D'Une Notice Historique et Chronologique des Travaux Imprimés Sur L'Art Du Dentiste Depuis Hippocrate Jusqu' A Nous, &c. &c. Deuxiéme Edition, pp. 839, 8vo. Paris, 1845.

*Complete Elements of the Science and Art of the Dentist,* followed by an Historical and Chronological Notice of works published on the Dental Art, from the time of Hippocrates, &c. &c. By M. Desirabode, *Surgeon Dentist to the King, assisted by his* Sons: Second Edition, pp. 839, 8vo. Paris, 1845.

We have not had time, since the receipt of the above named work, to give it a careful and attentive perusal, but from the parts of it which we have read, we feel warranted in pronouncing it a book of real merit. Its scope is as extensive as any other French treatise upon the same subject; and, while it contains much of the practical details of the profession, it, at the same time, treats to some considerable extent, on the physiology and pathology of the dental organism. The work comes to us, too, with a mark of approbation, which has seldom, if ever before, been bestowed upon a publication of this kind—it having been adopted, by ministerial ordinance, rendered upon the Report of the Royal Council of Public Instruction, for the Schools of Medicine and Pharmacy in France, as well as for the Hospitals of the Ports and Colonies of France, upon the Report of the Inspector General of the Marine Service of Health.

M. Desirabode, has long enjoyed a high reputation for skill as a practitioner of dental surgery, and although we are not so familiar with the professional reputation and abilities of his sons, we doubt not, from the thorough medico-dental education which they have received, they are well qualified for the part of the task, which was imposed upon them, in the preparation of the work under consideration.

The diseases of the dental organism, very properly occupy the most prominent place in the work, and from the nature of these, rather than from experience, they deduce the remedial indications. In this, M. M. Desirabode are correct, and it is only by perusing the same plan, that dental surgery can be elevated to a level with the other branches of the curative art, and practice, based upon a thorough knowledge of pathology, has always been proven by experience to be correct. But experienee alone, is not a safe nor an infallible guide, for the same results are not always obtained from the same practice. These are influenced by a thousand peculiarities and circumstances, which can only be properly understood by those who have made the nature and causes of disease a study.

The work is divided into two parts. The first treats of Anatomy, Physiology, Hygiene, Orthopedie and Therapeutics. The second part is devoted to the practical details of the art.

If time permitted, we should like to go into a critical analysis of the work, but for want of which, we must content ourself with a brief notice of some one or two of the subjects, embraced in it.

In the first place, then, we will notice his views with regard to the vitality of the teeth, which some very able writers deny, attributing the sensibility which they so frequently manifest, to the transmission of the impression to the pulp. M. Desirabode maintains the converse of this doctrine, and in support of the correctness of his opinion, he adduces the following experiment. He took a recently extracted sound tooth, introduced into its cavity a piece of wood dipped in blue turnsole, and then applied to its crown, by means of a camel hair pencil, an acid. Now he argues, and we believe with reason, if the peculiar sensation which usually results from the application of acids to the teeth, depended on the action of the acid on the dental pulp, which immediately imbibes all the ossiform part, this action would evidently have exerted itself on the blue color, with which the canal had been filled, and turned it red; but he says nothing of the sort took place, and that it was a long time before this color was attacked by the acid. He furthermore states, that he has held, immersed in vinegar, for several minutes, the crowns of teeth, whose canals he had previously filled with the syrup of violets, and that the color of the latter, had not been affected by it. From this, he infers, that the sensation must have its seat in parts not so deeply situated as the pulp.

The author's remarks on tooth-ache, are characterised, not only by good sense, but also by a knowledge of the nature and causes of the affection, and if we cannot fully subscribe to all of the views of M. Desirabode, upon this subject, we believe them, for the most part, to be correct.

In treating of the diseases of the gums, the authors describe an affection, which we believe, we were the first to notice.* It consists of an exudation of a purulent secretion, from between the edges of the gums, and necks of the teeth. At the time we wrote our "Principles and Practice of Dental Surgery," no mention, that we are aware of, had ever been made by any writer, of this disease.

The several varieties of disease to which the gums are liable, are treated of in the work before us, but we cannot, at present, give even an outline of the authors' views upon the subject.

In the treatment of lesions of the palatine organs, he recommends the employment of a description of obturators, which he thinks are calculated to favor the efforts of nature, for the reparation of the injury. The importance of leaving the parts free to contract, has been suggested by dental practitioners before, and hence the old method of confining obturators, by means of wings, pieces of sponge, &c. &c., were long since abandoned, and the more rational plan of securing them by clasps, fixed to the molar or bicuspid teeth, adopted. But M. Desirabode, not satisfied with leaving the restoration of the parts to the curative operations of the economy alone, he endeavors, while he closes the opening by means of a mechanical appliance, to aid the natural tendency of the aperture to contract, by the employment, in the construction of his artificial obturator, of caoutchouc. The contractility of this agent, we have no doubt, may be made available, in assisting the contraction of the soft parts, but we doubt whether it can be made to effect any salutary change, on the solid tissues, as is supposed by M. Desirabode.

In conclusion, it may be proper to remark, that so far as the plan of this work is concerned, it possesses no claim to novelty, and while as a whole, we regard it as one of the best French works upon dental surgery that has ever been published upon the subject, we do not wish to be understood as considering it faultless, or as containing all which it is necessary for the dentist to know. The work, however, notwithstanding it contains doctrines both

* Vide Principles and Practice of Dental Surgery.

theoretical and practical, which we regard as evidently erroneous, possesses such high claims to consideration, we cannot refrain from awarding to the accomplished authors, the high meed of praise, to which we think them so justly entitled. It is well written, and is entitled to a high place in the literature of dental surgery.—*Balt. Ed.*

*Réflexions sur les Moyens Employés Jusq'a ce Jour Pour le Redressement des Dents Suivies de la Description, d'un Procédé Nouveau.* Par M. P. A. Grandhomme, Chirurgien Dentiste, 4to. pp. 20. Paris, 1845.

*Reflections on the Means Employed at the present day, for Regulating the Teeth, with a Description of a New Method of Procedure.* By M. P. A. Grandhomme, Surgeon Dentist, 4to. pp. 20. Paris, 1845.

The above is the title of an Essay, which we have but very recently received, and have not yet had leisure to read it. It is printed on beautiful paper, and in every respect, neatly gotten up.—*Balt. Ed.*

*L'Encyclopédie du Dentiste,* ou Repertoire Général de Toutes les Connaissances Medico-Chirurgicales sur L'Anatomie et la Pathologie des Dents, sur les Deux Dentitions; Avec Counseils Aux Méres, Aux Nourrices et Aux Gens du Monde, sur les Soins de la Bouche et les Moyens de Conserver les Dents Saines et Bells. Pricédé de L'Histoire du Dentiste chez les Anciens. Et Accompagné D'un Traite Complet sur les Dents Artificielles, et principalement sur les Osanores. Par William Rogers, Dentiste, 8vo. pp. 470. Paris, 1845.

*Encyclopedia of Dentistry,* or General Repertory of all the Medico-Chirurgical knowledge upon the Anatomy and Pathology of the Teeth; the two Dentitions, with advice to mothers, nurses and people generally, on the care of the mouth, and the means for preserving the teeth in health and beauty. Preceded by the History of Dentistry among the Ancients; and accompanied by a complete Treatise on Artificial Teeth, and particularly upon *Osanores.* By William Rogers, Dentist, 8vo. pp. 470. Paris, 1845.

If this work was, indeed, what it professes to be, it would be an acquisition to the literature of the dental branch of medicine, and if the author, who by-the-by, is an Englishman, intended to make it, what its title proclaims it to be, we can only say that he has wholly failed in the accomplishment of his design. It is any thing else than a repertory of the medico-chirurgical knowledge of dental surgery, or a history of the art among the ancients. Nor is it a complete treatise on artificial teeth, or even upon his vaunted "*Osanores,*" a name which the author has given to a description of artificial teeth, of which he claims to be the inventor, and which he pronounces superior to any other ever employed.

If we were disposed to award to the author of the Encyclopedia of Dentistry, any praise, he has rendered such commendation unnecessary, by the profuse manner in which he has bestowed it upon himself. Judging from the extravagant manner in which he compliments his own *unequalled* abilities and wonderful performances, we should be inclined to believe him, at least, a thousand years in advance of the profession, as practised even by the most skilful, except, of course, *by himself.*

The work is characterised by the most fulsome boasting. The triumphs of his own superior system of practice over all others, and the envy of other practitioners, are set forth in a strain of grandiloquence, which, if not amusing, is, at least, well calculated to inspire the reader with the most hearty disgust for the author. We think it a pity that so much good paper should be spoiled with such miserable trash as is contained in the volume before us. Should the author ever acquire a scientific and thorough knowledge of the profession which he professes to love so much, he will doubtless regret ever having sent into the world such a palpable evidence of his ignorance and folly.

The following translation of a passage will enable the reader to form some idea of the author's modesty. In speaking of his *osanores,* he says, "Like all innovators, I had at first to contend against the prejudice of routine; my confreres vehemently opposed *my system,* and I had, for a long time, to sustain violent opposition. I have not lost courage, and sure of the efficacy of my invention, I have triumphed over all obstacles, because good and great things always triumph over error and even jealousy. I have reduced my antagonists to silence, by opposing to their invectives cures which were regarded hopeless, results which pertained to the miraculous. While my means were very simple, I endeavored to imitate nature in repairing disasters caused by the numerous diseases to which the buccal organs are subject."

Again, a little farther on, he says, "I can affirm, with that boldness which a consciousness of having rendered a service to my species inspires, that I have effected a complete revolution in the dental art, which will have its course, and change the character of dental surgery."

Now we should like to tell our readers in what his *great* invention consists; but not being able to find out, from any description he has given of it, we are unable to do so. The only clue to it which we can obtain from the work, is derived from the circumstance which he mentions, near the conclusion of the book, that induced him to give to it the name *osanores.* It was because the teeth were retained in the mouth without the aid of ligatures, clasps or gold wire, and from this it would seem that it consists in the application of artificial teeth upon what is termed the atmospheric pressure or suction principle, which has been practiced in this country and many parts of Europe for a number of years.—*Balt. Ed.*

